# Data of *in vivo* screening of antiulcer activity for methanolic extract of *Vernonia elaeagnifolia* DC

**DOI:** 10.1016/j.dib.2019.103753

**Published:** 2019-03-07

**Authors:** Sneha Nawale, N. Priyanka, Sujit Das, M. Ganga Raju

**Affiliations:** Department of Pharmacognosy, Gokaraju Rangaraju College of Pharmacy, Bachupally, Hyderabad 500090, India

**Keywords:** Antiulcer activity, *Vernonia elaeagnifolia*, Omeprazole, Ethanol, Aspirin, Pylorus ligation, MEVE, Methanolic extract of *Vernonia elaeagnifolia*, NO, nitric oxide, IAEC, Institutional Animal Ethical Committee, H_2_O_2_, Hydrogen peroxide, CPCSEA, Committee for the purpose of control and supervision of experimentation on animals, OECD, Economic Cooperation and Development, *p.o*, per oral, ANOVA, Analysis of variance, bd.wt, body weight, SEM, standard error of mean, mg/kg, Milligram per Kilogram, LD_50_, Median lethal dose, mEq, Milliequivalents, mg, Milligram, mL, Millilitre, %, percentage, SEM, standard error of mean

## Abstract

The data present in this article is related to evaluation of standardized methanolic extract of *Vernonia elaeagnifolia* aerial parts [MEVE]*,* a species of Asteraceae family for antiulcer potential. Antiulcer activity of MEVE (200 and 400 mg/kg, b.w., *p.o.*) was evaluated with ethanol and aspirin induced ulcer models and pylorus ligation induced gastric ulcer model. The antioxidant potential of MEVE was evaluated with nitric oxide radicals, hydroxyl radical and H_2_O_2_ radical scavenging assay against standard ascorbic acid to correlate antioxidant and antiulcerogenic action. MEVE significantly protects the gastric mucosa against the ethanol and aspirin induced ulcer and pylorus ligation induced ulcer challenge. MEVE had shown significant [normal control: p < 0.0001, disease control: p < 0.0001, standard: p < 0.0001] decrease in the ulcer index produced by all three models in rats as compared to the standard drug omeprazole [20 mg/kg, b.w., *p.o.*].

The present data suggest that aerial parts of *Vernonia elaeagnifolia* possess significant antiulcer activity, which may attributed to its antioxidant mechanism of action.

**Table:** Specifications table

Subject area	*Pharmacy*
More specific subject area	*Antiulcer activity of medicinal plant.*
Type of data	*Table, text file, graph, figure.*
How data was acquired	*Histopathology study of rat's stomach mucosal layer was performed for aspirin induced ulcer model (disease induced, MEVE treated, and standard groups).*
Data format	*Analysed*
Experimental factors	*Methanolic extract of Vernonia elaeagnifolia used for present study was prepared by using soxhlet extraction method.* *1. Acute toxicity study was performed with female mice followed by OECD guideline 423.* *2. Antiulcer activity was performed for MEVE by ethanol and aspirin induced ulcer models along with pylorus ligation.* *Rats were divided into five groups of six rats (n* = *6) each. Group I served as positive control and Group II disease control (negative control), Group III and IV, were treated with MEVE at doses of 200 and* 400 mg/Kg bd*. Wt. respectively and Group V was treated with standard.*
Experimental features	*Ulcer index and percentage ulcer protection was calculated in ethanol, aspirin induced ulcer models and pylorus ligation model and pH, total acidity and free acidity was estimated in pylorus ligation model for MEVE at 200 and* 400 mg/kg*, bd.wt, p.o .*
Data source location	*Department of pharmacology,* *Gokaraju Rangaraju college of pharmacy,* *Bachupally, Hyderabad-500090, Telangana.*
Data accessibility	*All data are presented in the article*
Related research articles	*1. Jyoti Gupta, Dinesh Kuma, Ankit Gupta. Evaluation of gastric anti-ulcer activity of methanolic extract of Cayratia trifolia in experimental animals. Asian Pac J Trop Dis 2012; (12):99–102.*
	*2. Manoj Kumar Choudhary, Surendra H. Bodakhe, Sanjay Kumar Gupta. Assessment of the Antiulcer Potential of Moringa oleifera Root-Bark Extract in Rats. J Acupunct Meridian Stud 2013; 6(4):214–220.*
	*3. R. Sathish, Bhushan Vyawahare, K. Natarajan Antiulcerogenic activity of Lantana camara leaves on gastric and duodenal ulcers in experimental rats. J Ethnopharmacol 2011; 134:195–197.*

**Table:** 

**Value of the data** Antiulcer activity of *Vernonia elaeagnifolia* is recognized due to; • Plants of Astraceae family have medicinal properties against upper respiratory tract infections, stomach ulceration and skin infections. V. elaeagnifolia extract showed presence of various phytoconstituents as flavonoids, phenolic compounds, terpenoids, phytosterols, and coumarins, which are previously proved to possess antiulcer activity. • The methods and data can be used to study Vernonia elaeagnifolia for its antiulcer potential in detail. • Data demonstrates the comparison of antiulcer activity of MEVE at the doses 200 and 400 mg/kg bd.wt. p.o. with marketed formulation, gives reference for researchers to study the formulation.

## 1 Data

**Fig. 1:**
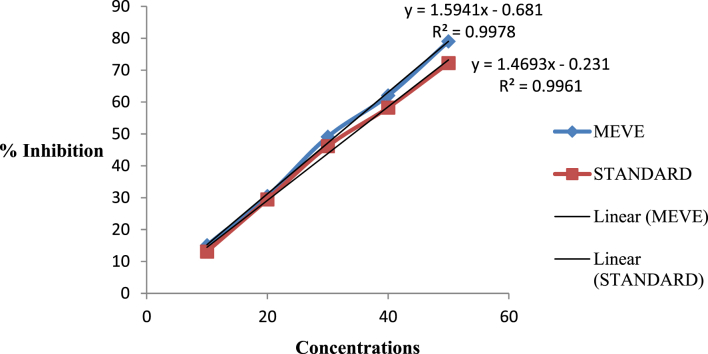
Effect of MEVE and catechol (standard) on nitric oxide radical scavenging assay.

**Fig. 2:**
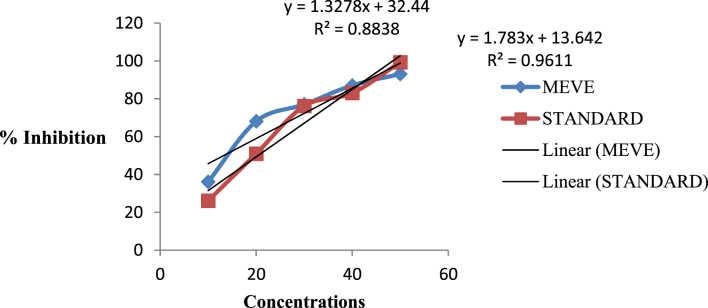
Effect of MEVE and ascorbic acid (standard) on hydroxyl radical scavenging assay.

**Fig. 3:**
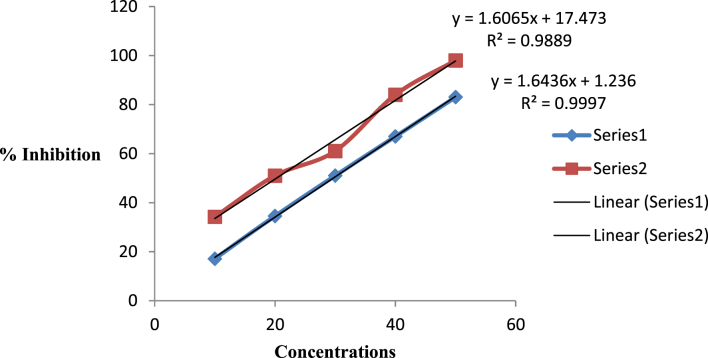
Effect of MEVE and ascorbic acid (standard) on hydrogen peroxide radical scavenging assay.

**Fig. 4:**
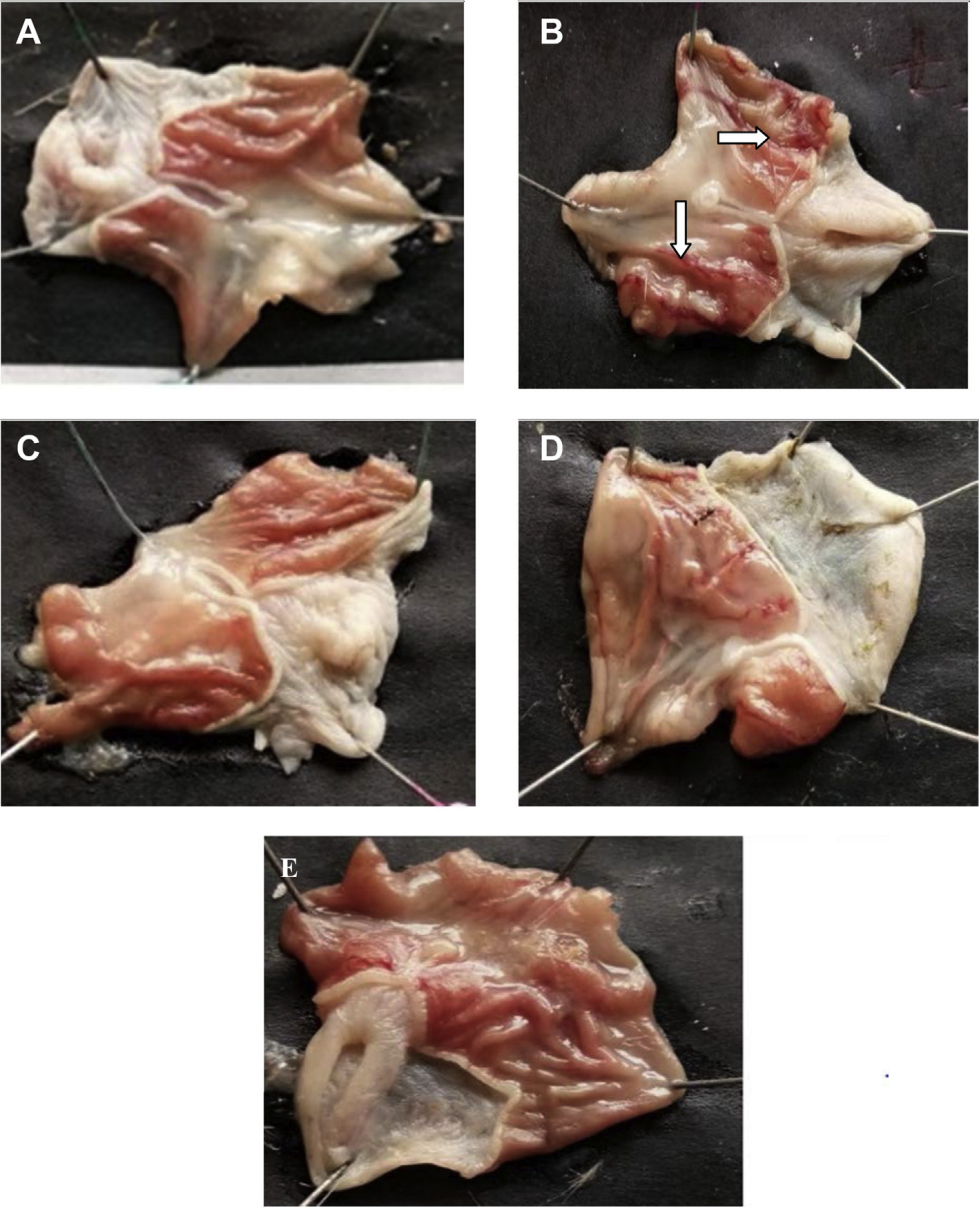
**Macroscopic appearance of the gastric mucosa in ethanol induced ulcers in rat**. A. Group-1: Normal control, B. Group-2: Disease induced (Streaks was observed), C. Group-3: (T_1_) [MEVE] (200 mg/kg,bd.wt,*p.o*), D. Group-4: [MEVE] (400 mg/kg,bd.wt,*p.o*), E. Group-5: Standard [Omeprazole] (20 mg/kg,bd.wt,*p.o*).

**Fig. 5:**
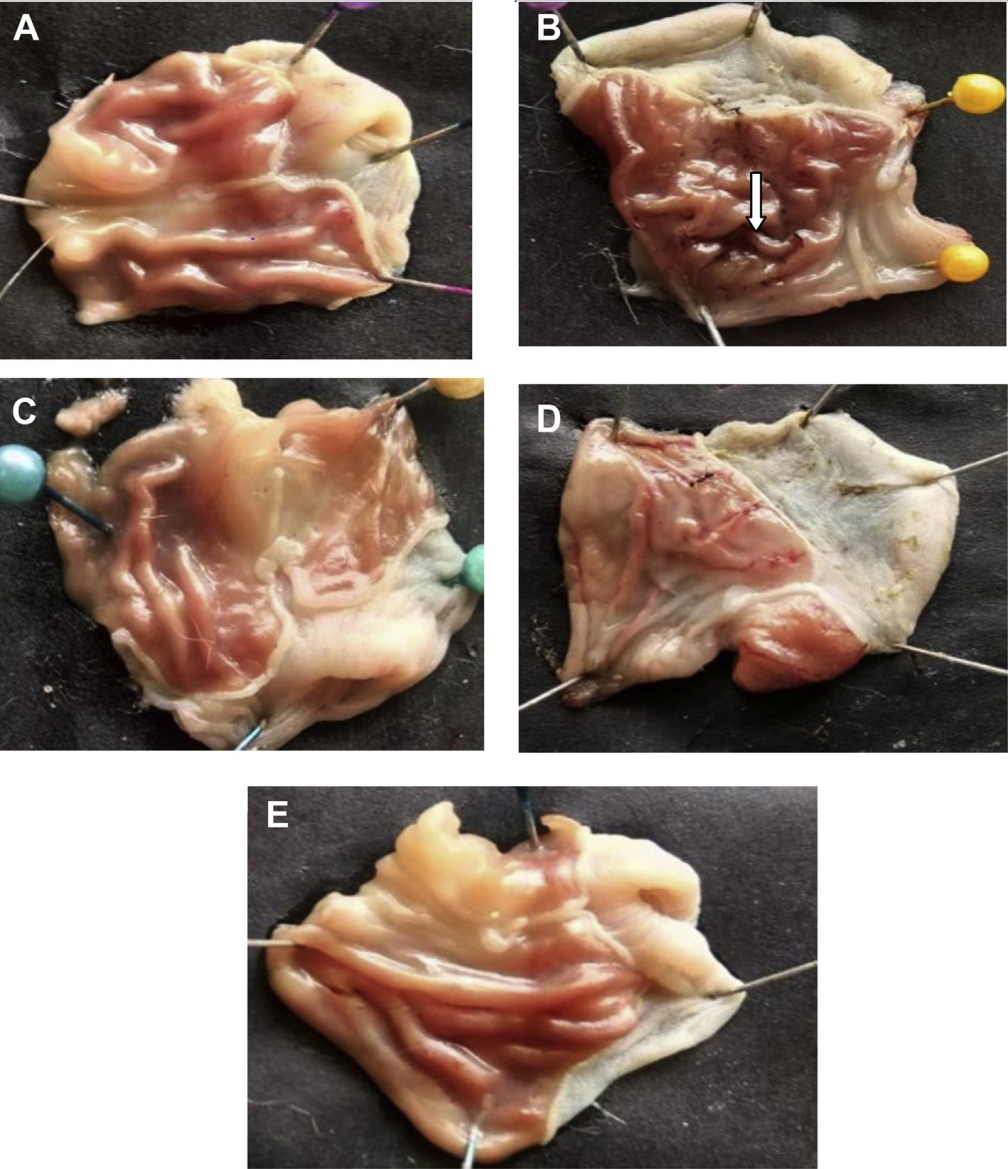
**Macroscopic appearance of the gastric mucosa in aspirin induced ulcer model**. A. Group -1: Normal control, B. Group -2: Disease control (Perforations was observed), C. Group– 3: [MEVE] (200 mg/kg,bd.wt,*p.o*), D. Group – 4: [MEVE] (400 mg/kg,bd.wt,*p.o*), E. Group – 5: Standard [Omeprazole] (20 mg/kg,bd.wt,*p.o*).

**Fig. 6:**
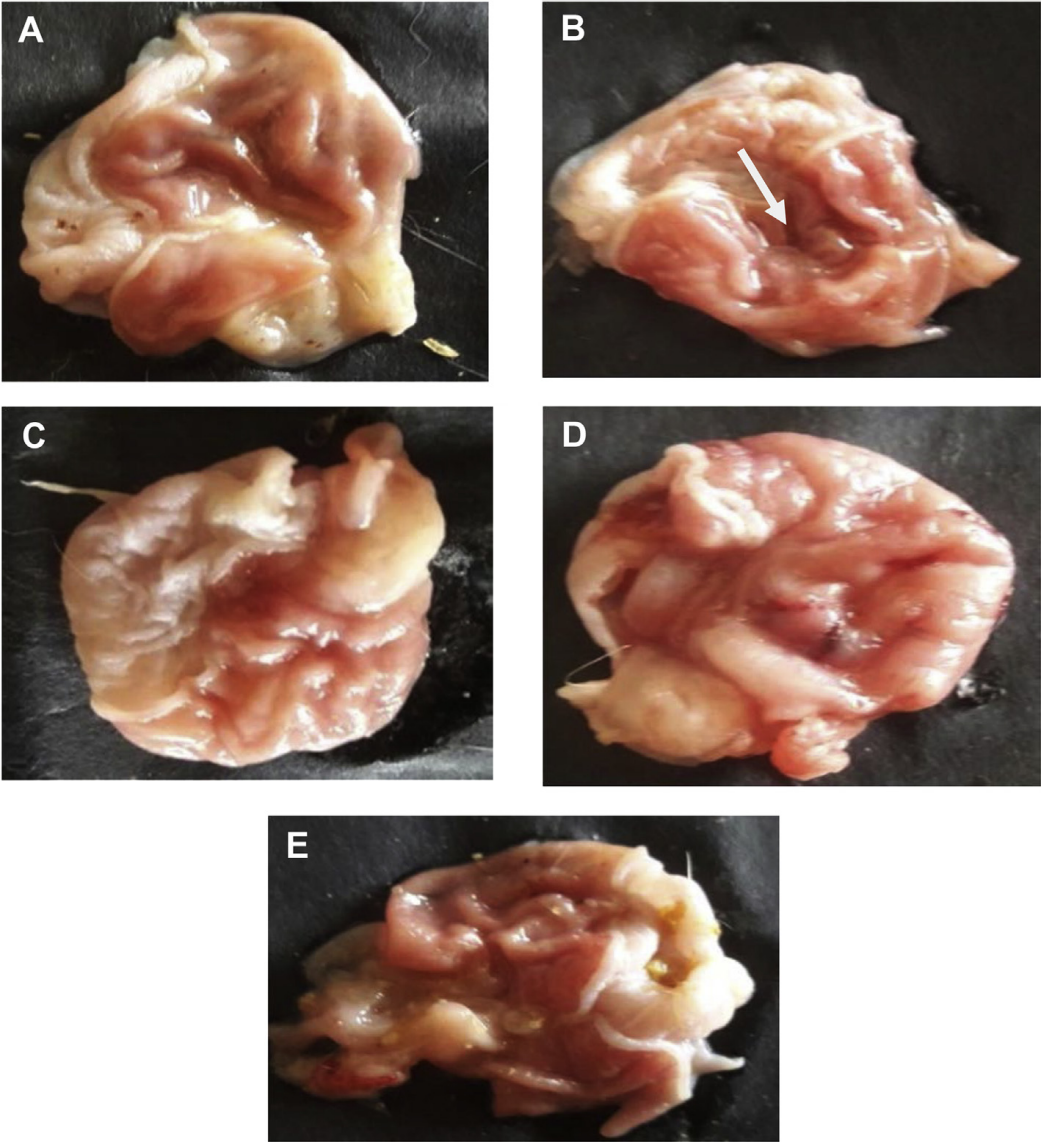
**Macroscopic appearance of the gastric mucosa in pylorus ligation model**. A. Group -1: Normal control, B. Group -2: Disease control (Perforations was observed), C. Group– 3: [MEVE] (200 mg/kg,bd.wt,*p.o*), D. Group – 4: [MEVE] (400 mg/kg,bd.wt,*p.o*), E. Group – 5: Standard [Omeprazole] (20 mg/kg,bd.wt,*p.o*).

**Fig. 7:**
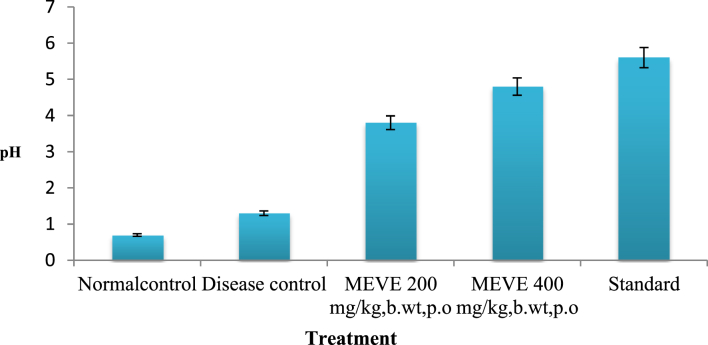
Effect of MEVE on gastric pH in pylorus ligated gastric ulcer.

**Fig. 8:**
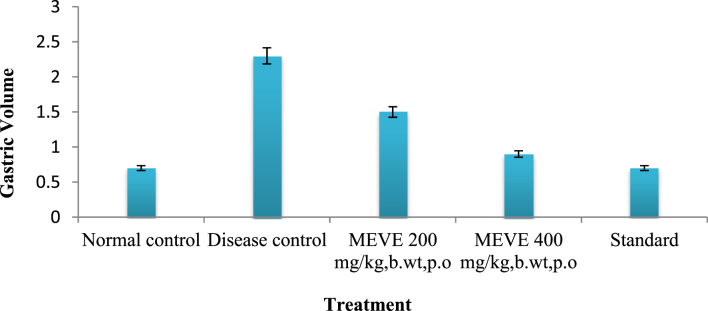
Effect of MEVE on gastric volume in pylorus ligated gastric ulcer.

**Fig. 9:**
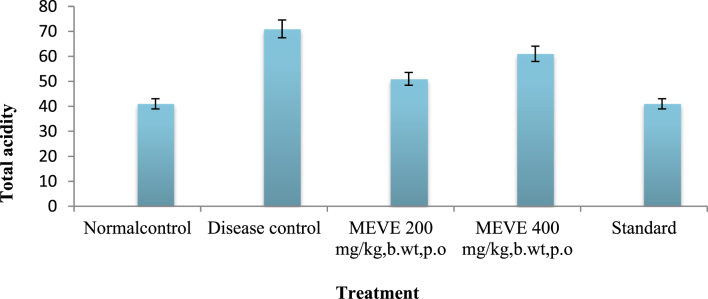
Effect of MEVE on total acidity in pylorus ligated gastric ulcer.

**Fig. 10:**
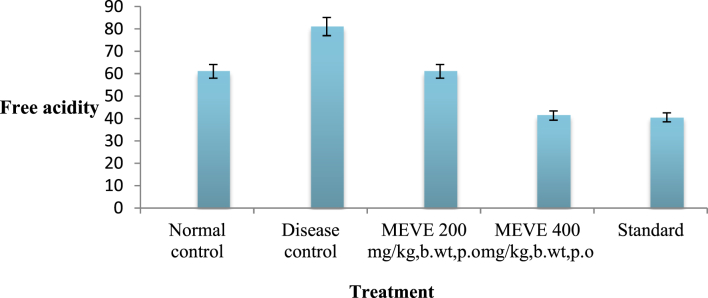
Effect of MEVE on free acidity in pylorus ligated gastric ulcer.

**Fig. 11:**
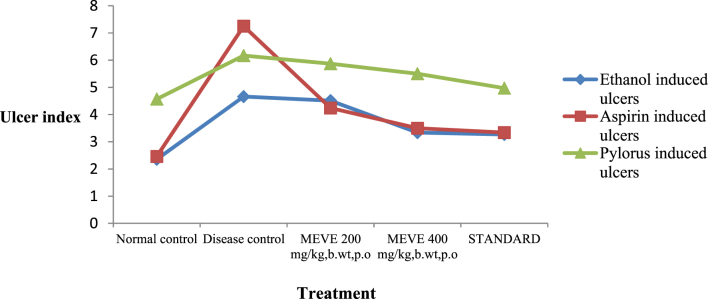
Effect of MEVE on ulcer index by ethanol induced ulcer, aspirin induced ulcer and pylorus ligation induced ulcers in rats.

**Fig. 12:**
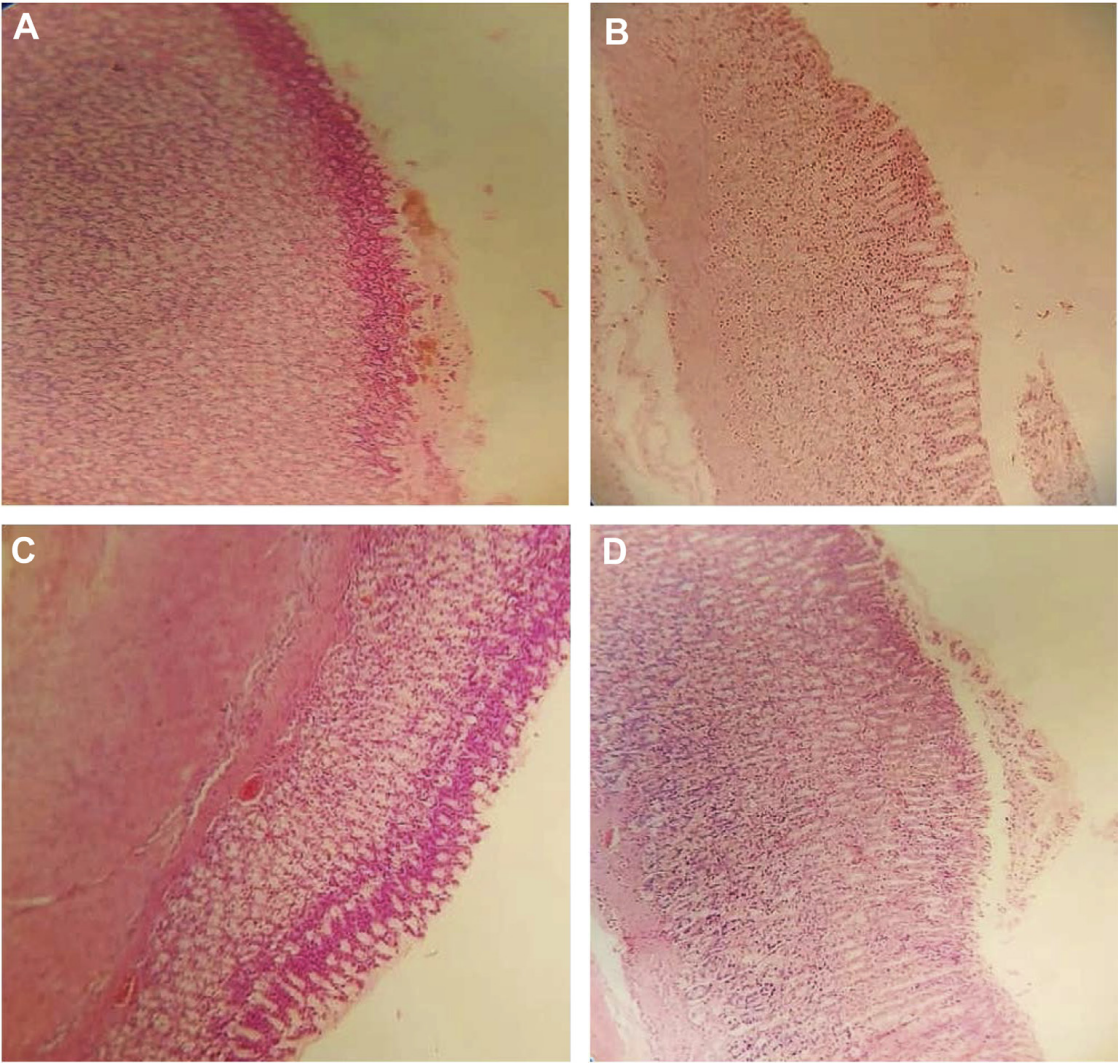
**Histopathology of the stomach mucosal of aspirin induced ulcers in rats.** A. Histopathology of rats in Disease control (negative control) group, Gastric mucosal hyperplasia is noted, Gastric pits or foveolar decreased in size, and inflammation was also observed. B. In MEVE treated group at dose of 200 mg/kg, bd.wt, *p.o,* Gastric mucosal appeared to be normal, scant inflammatory cells appeared to be normal, No hyperplasia was observed. C. In MEVE treated group at a dose of 400 mg/kg, bd.wt, *p.o,* Scant inflammatory cells appear normal, Gastric mucosal thickness appeared to be normal, Slight hyperplasia was observed. D. In standard treated group, Omeprazole at a dose of 20 mg/kg, bd.wt, *p.o* showed Normal foveolar, Mucosal thickness appeared to be normal, No inflammation was observed.

The present data focus on evaluating the antiulcer activity of methanolic extract of *V. elaeagnifolia* (family Asteraceae). *V. elaeaegnifolia* was widely known various medicinal properties and also studied for its traditional uses against upper respiratory tract infections, stomach ulceration, skin infections and as leech repellent [1]. MEVE showed prominent radical scavenging action for nitric oxide, hydroxyl and hydrogen peroxide radical. Data is presented in Table 1 (Fig. 1, Fig. 2, Fig. 3). MEVE has shown significant reduction in ulcer index and percentage inhibition of ulcer formation in ethanol and aspirin induced ulcer, along with pylorus ligation method. Data is presented in Table 2, Table 3, Table 4 (Fig. 4A–E, Fig. 5A–E, and Fig. 6A–E) and effect of extract on ulcer healing study, presented in Table 5 (Fig. 7, Fig. 8, Fig. 9, Fig. 10 and Fig. 11). Data regarding histological changes in mucosal layer of rat stomach for aspirin induced model are shown in (Fig. 12A–D).

**Table 1:** Antioxidant assay of methanolic extract of *V. elaeagnifolia*.

S. No	Assay	Test compound	IC_50_ value _(μg/mL)_
1	Nitric oxide radical scavenging assay	MEVE	34.18
		Standard (catechol)	31.79
2	Hydroxyl radical scavenging assay	MEVE	20.91
		Standard (Ascorbic acid)	13.22
3	Hydrogen peroxide radical scavenging assay	MEVE	29.22
		Standard (Ascorbic acid)	20.24

**Table 2:** Effect of methanolic extract of *V.elaeagnifolia* in ethanol induced acute gastric ulcers rats.

Group	Treatment	Ulcer Index	% Biological activity	% relative activity
Group-I	Normal control (positive control)	2.35±0.01	–	
Group-II	Disease induced (1mL/200g)(Negative control)	4.66±0.01	–	
Group-III	MEVE(200 mg/kg, bd.wt.,*p.o*)	4.51±0.01**^A,a^	3	8.034
Group-IV	MEVE (400 mg/kg,bd.wt.,*p.o*)	3.39±0.01**^A,a^	27	4.854
Group-V	Omeprazole (20 mg/kg, bd.wt., *p.o)*	3.32±0.01**^,a^	29	

Values are expressed as mean ± SEM, (n = 6). Statistical analysis was performed by using ANOVA by Dunnett's test. Results were compared Normal control [P < 0.0001**] Disease control [P < 0.0001^a^] Standard [p < 0.0001^A^].

**Table 3:** Effect of methanolic extract of *V.elaeagnifolia* in aspirin induced gastric ulcers in rats.

Group	Treatment	Ulcer index	% Biological activity	% relative Activity
Group-I	Normal control (positive control)	2.46 ± 0.01	–	
Group-II	Disease control (Negative control)	7.25 ± 0.01	–	
Group-III	MEVE (200 mg/kg, bd.wt, *p.o*)	4.24 ± 0.01**^A,a^	41	8.48
Group-IV	MEVE (400 mg/kg, bd.wt, *p.o*)	3.5 ± 0.01**^A,a^	51	4.89
Group-V	Omeprazole (20 mg/kg, bd.wt, *p.o*)	3.34 ± 0.01**^,a^	53	

Values are expressed as mean ± SEM, (n = 6). Statistical analysis was performed by using ANOVA by Dunnett's test. Results were compared Normal control [P < 0.0001**] Disease control [P < 0.0001^a^] Standard [p < 0.0001^A^].

**Table 4:** Effect of methanolic extract of *V.elaeagnifolia* in pylorus ligation induced gastric ulcers in rats.

Group	Treatment	Ulcer index	% Biological activity	% relative Activity
Group–I	Normal control (positive control)	4.57 ± 0.03	–	
Group-II	Disease control (Negative control)	6.17 ± 0.01	–	
Group-III	MEVE (200 mg/kg, bd.wt, *p.o*)	5.87 ± 0.01**^A,a^	4	7.88
Group-IV	MEVE (400 mg/kg, bd.wt*, p.o*)	5.50 ± 0.01**^A,a^	10	4.46
Group-V	Omeprazole (20 mg/kg, bd.wt, *p.o)*	4.97 ± 0.01**^,a^	19	

Values are expressed as mean ± SEM, (n = 6). Statistical analysis was performed by using ANOVA by Dunnett's test. Results were compared with NC [p < 0.0001**], DI [p < 0.0001^a^], Standard [p < 0.0001^A^].

## 2 Experimental design, materials and methods

### 2.1 Plant collection and extraction

Aerial parts of *V. elaeagnifolia* were collected, during the month of January 2018 from R.R district, Hyderabad, Telangana. The plant was identified and authenticated (Voucher specimen no., VEN-3) by Botanist Dr. Rabiya sultana, Junior Lecturer, New Government Junior College, kukatpally, Hyderabad.

### 2.2 Chemicals and reagents

Aspirin used in study was procured from Reckitt Benckiser and Omeprazole from Alkem Laboratories.

### 2.3 Preparation of extract

#### 2.3.1 Plant extract

The aerial parts of *V. elaeagnifolia* were cleaned, dried under shade for about ten days and coarsely powdered in a pulveriser. The powdered material was taken up for soxhlet extraction process. The crude powdered drug (500 g) was extracted with 90% methanol (1500 mL) by soxhlation.

### 2.4 Preliminary phytochemical screening

Preliminary phytochemical screening of crude extract was performed by various chemical tests to identify various phytoconstituents like flavonoids, tannins and phenolic compounds, alkaloids, terpenoids [2].

### 2.5 *In vitro* antioxidant assay of MEVE

Nitric oxide, hydroxyl and hydrogen peroxide radicals are potent reactive oxygen species in the biological system that reacts with polyunsaturated fatty acid moieties of the cell membrane phospholipids and causes damage to the cell leading to various chronic diseases. The scavenging ability of MEVE for nitric oxide, hydroxyl and hydrogen peroxide radicals was measured by the method of Kunchandy and Rao (1990) [3].

In nitric oxide scavenging assay, 2 mL of 10 mM sodium nitroprusside dissolved in 0.5 mL phosphate buffer (pH 7.4) and mixed with 0.5 mL of MEVE at various concentrations (10, 20, 30, 40, 50 μg/mL) and ascorbic acid (10, 20, 30, 40, 50 μg/mL). The resultant mixture was then incubated at 25 °C for 150 min. After incubation, 0.5 mL of the incubated solution was mixed with 0.5 mL of Griess reagent. The mixture was again incubated at room temperature for 30 min and absorbance was measured at 546 nm [4].

In hydroxyl radical scavenging assay, the reaction mixture was prepared by adding 100 μL of 2-deoxy- D ribose (28 mM in 20 mM KH_2_PO_4_—KOH buffer, pH 7.4), 500 μL of MEVE at different concentrations (10, 20, 30, 40, 50 μg/mL), 200 μL EDTA (1.04 mM) and 200 μM FeCl_3_, 100 μL of H_2_O_2_ (1 mM) and 100 μL ascorbic acid (1mM), and incubated at 37 °C for 1 h. 1mL thiobarbituric acid (1%) and 1mL of trichloroacetic acid (2.8%) was added to resultant mixture and again incubated at 100 °C for 20 min. After cooling, absorbance of resultant solution was measured at 532 nm, against a blank sample [5].

In hydrogen peroxide radical scavenging assay, hydrogen peroxide solution (40 mM) was prepared in phosphate buffer (pH 7.4). 4 mL of MEVE at different concetrations (10, 20, 30, 40, 50 μg/mL) was added to a hydrogen peroxide solution (0.6 mL, 40mM). Absorbance of resultant solution was measured at 230 nm after 10 minutes against a blank solution solution [6]. Data outcome is shown in Table -1 (Fig. 1, Fig. 2, Fig. 3).

### 2.6 Animals

Albino rats of Wistar strain of either sex weighing between 170 and 200 g were used; Animals were procured from Jeeva life sciences, Hyderabad, T.S. They were housed in standard cages at room temperature (25 ± 2 °C) and provided with pellet diet procured from Albino labs, Hyderabad and water *ad libitum*. The animals were deprived of food for 24 h before experimentation, but had free access to drinking water. The study was conducted after obtaining institutional ethical committee clearance bearing the number 1175/Po/Re/s/08/CPCSEA.

### 2.7 Acute toxicity studies

An acute toxicity study was carried out in order to check the toxic effects for methanolic extract of *V. elaeagnifolia* on female mice (This is because literature surveys of conventional LD _50_ tests show that generally females were slightly more sensitive and single sex of animals is used in order to reduce variability and means of minimizing the number of animals used). The study was performed as per Organization for Economic Cooperation and Development (OECD) and acute oral toxicity was done by up and down procedure (OECD guideline-425) [7].

### 2.8 Evaluation of anti-ulcerogenic activity of MEVE on rats

#### 2.8.1 Ethanol induced ulcers model in rats

**Table:** 

Group –I	Normal control (Positive control) received vehicle
Group-II	Disease control (Negative control) received ethanol 90% (5 mL/kg, bd.wt*, p.o)* on the day of experiment i.e. 10th day.
Group-III	Received MEVE (200 mg/kg, bd.wt, *p.o*) for 10 days + on 10 ^th^ day after final dose, received ethanol 90% (5 mL/kg.*,*bd.wt, *p.o)*
Group-IV	Received MEVE (400 mg/kg, bd.wt, *p.o*) for 10 days + on 10 ^th^ day after final dose, received ethanol 90% (5 mL/kg.,bd.wt, *p.o*)
Group-V	Standard omeprazole (20 mg/kg.,bd.wt, *p.o)* + received ethanol 90% (5 mL/kg.*,*bd.wt, *p.o)* on the day of experiment.

Group I and II received vehicle (10 day), Group III and IV received MEVE 200 and 400 mg/kg, b.wt, *p.o* respectively and Group V received omeprazole (20 mg/kg, b.wt, *p.o* with stainless steel gavage needle) for 10 days. On 10th day, 1 h after the final dose of treatment, 90% ethanol (5 mL/kg, b.wt, *p.o.* with stainless steel gavage needle) was administered to overnight fasted rats of all groups other than normal control (positive control). The animals were sacrificed after 1 h of ulcerogen (ethanol) administration and their stomach will be excised and cut opened along the greater curvature to determine ulcer index. Ulcer areas on the stomach's surface were examined macroscopically and measured on millimetre-square paper. The sum of area (mm^2^) was expressed as ulcer index (UI) and percentage inhibition was calculated [8].

#### 2.8.2 Evaluation of ulcer index

UI = 10/X

10 = factor for calculating ulcer index; X = total mucosal area/ total ulcerated area.

#### 2.8.3 Determination of percentage biological activity (% protection protection)

$$\%\;\text{biological}\;\text{activity}=\frac{\left[\text{control}\;\text{UI}\right]-\left[\text{test}\;\text{mean}\;\text{UI}\right]\times 100}{\left(\text{Control}\;\text{mean}\;\text{ulcer}\;\text{index}\right)}$$

UI= Ulcer index

Percent relative activity was calculated by formula

$$\%\;\text{relative}\;\text{activity}=\frac{\text{Biological}\;{\text{activity}}_{\text{test}\;\text{extract}}\times {\text{Dose}}_{\text{standard}}\times 100}{\text{Biological}\;{\text{activity}}_{\text{standard}}\times {\text{Dose}}_{\text{test}\;\text{extract}}}$$

### 2.9 Aspirin induced ulcers model in rats

**Table:** 

Group –I	Normal control (positive control) received vehicle
Group-II	Disease control (negative control) (fasting for 24 h + receive aspirin 200 mg/kg, bd.wt, *p.o)* on the day of experiment i.e. on 7th day.
Group-III	Received MEVE (200 mg/kg*., p.o)* for 7 days + on 7th day received aspirin (200 mg/kg, bd.wt, *p.o).*
Group-IV	Received MEVE (400 mg/kg, bd.wt., *p.o)* for 7 days + on 7 ^th^ day received aspirin (200 mg/kg, bd.wt.,*p.o*).
Group-V	Standard omeprazole (20 mg/kg, bd.wt, *p.o)* + on 7th day received aspirin (200 mg/kg, bd.wt, *p.o*).

Group I and II received vehicle, Group III and IV received MEVE (200 and 400 mg/kg, b.wt, *p.o.*) respectively and Group V received omeprazole (20 mg/kg, b.wt, *p.o*) for 7 days. On 7th day, Aspirin (200 mg/kg, bd.wt, *p.o.*) was administered to the all animals other than normal group with prior fasting of 24 h. The animals were sacrificed 4 h after administration of aspirin and the stomach part was excised, cut along the greater curvature, washed carefully with 5.0 mL of 90% NaCl and ulcer areas on the stomach's surface were examined macroscopically and measured on millimetre-square paper. The sum of area (mm^2^) was expressed as UI and percentage inhibition was calculated [9].

### 2.10 Pylorus ligation induced ulcers in rats

**Table:** 

Group –I	Normal control (positive control) received vehicle
Group-II	Disease control (negative control)
Group-III	Received MEVE (200 mg/kg*,* bd.wt*., p.o)*
Group-IV	Received MEVE (400 mg/kg, bd.wt., *p.o)*
Group-V	Standard omeprazole (20 mg/kg*,* bd.wt*., p.o)*

Albino wistar rats were fasted for 24 h with water ad *libitum*. Group-I and Group-II received distilled water, Group-III and Group-IV received MEVE 200 and 400 mg/kg, bd.wt, *p.o.* and Group-V received Omeprazole (20 mg/kg, bd.wt, *p.o.*) as a standard. After 1h of treatment, animals were anaesthetized with anesthetic ether; the abdomen was opened by a small midline incision below xiphoid process. Pyloric portion of the stomach was lifted out and ligated according to method of Shay et al., 1945 [10] avoiding traction to the pylorus or damage to its blood supply. The stomach was replaced carefully and the abdominal wall was closed by interrupted sutures. Rats were sacrificed by CO_2_ euthanasia after 4 h of pyloric ligation. The abdomen was opened, cardiac end of the stomach was dissected out and the contents were drained into a glass tube. The volume of the gastric juice was measured and centrifuged at 2000 rpm for 10 min. From the supernatant, aliquots (1 mL of each) were taken for the determination of pH, total and free acidity. Ulcer areas on the stomach's surface were examined macroscopically and measured on millimetre-square paper. The sum of area (mm^2^) was expressed as UI and percentage inhibition was calculated [11], [12], [13].

#### 2.10.1 Determination of pH

An aliquot of 1mL gastric juice was diluted with 1mL of distilled water and pH of the solution was determined using pH meter (LI-617, Digital pH Meter, Elico).

#### 2.10.2 Determination of total acidity

An aliquot of 1mL gastric juice was diluted with 1mL of distilled water and taken into a 50 mL conical flask, two drops of phenolphthalein indicator was added to it and titrated with 0.01N NaOH until a permanent pink colour was observed. The volume of 0.01N NaOH consumed was noted. The total acidity is expressed as mEq/L by the following formula:

$$\text{Activity}=\frac{\text{Vol.}\;\text{of}\;\text{NaOH}\times \text{N}\times 100\;\text{mEq}/\text{L}}{0.1}$$

#### 2.10.3 Determination of free acidity

Aliquot of gastric juice was titrated with 0.01N NaOH by adding Topfer's reagent as an indicator until a permanent pink colour was observed. The volume of 0.01N NaOH consumed was noted and free acidity was calculated by the same formula for the determination of total acidity.

### 2.11 Ulcer healing study

**Table 5:** Effect of MEVE pretreatment on pylorus ligation-induced gastric ulcer.

S. NO	Groups	pH	Gastric volume	Free acidity	% relative Activity	Total acidity	% relative Activity
1	Control (positive control)	0.7 ± 0.1	1.3 ± 0.1	61 ± 1.52		41 ± 1.52	
2	Disease (Negative) control	1.3 ± 0.1	2.3 ± 0.1	81 ± 1.54		71 ± 1.57	
3	MEVE (200 mg/kg, bd.wt, *p.o*)	3.8 ± 0.1**^A,a^	1.5 ± .1**^A,a^	61 ± 1.56**^A,a^	15.06	51 ± 1.56**^A,a^	12.43
4	MEVE (400 mg/kg, bd.wt*, p.o*)	4.6 ± 0.1**^A,a^	0.9 ± 0.11^ns^	41.3 ± 1.58 ^ns^	5.098	61 ± 1.52**^A,a^	7.43
5	Standard	5.6 ± 0.1**^,a^	0.7 ± 0.1**^,a^	40.5 ± 1.54 **^,a^		41 ± 1.54**^,a^	

Values are expressed as mean ± SEM, (n = 6). Statistical analysis was performed by using ANOVA by Dunnett's test. Results were compared NC [P < 0.0001**] DI [P < 0.0001 a] Standard [p < 0.0001A], [ns = non significant].

### 2.12 Histopathology of the stomach mucosal of aspirin induced ulcers in rats

On 7th days of study, rats were sacrificed by CO_2_ euthanasia, to separate stomach and fixed in 10% formalin for 24 h, and gave for histopathological studies (Varun histopath, Hyderabad).

### 2.13 Statistical analysis

The results were expressed as mean SEM from six animals. The results were subjected to statistical analysis by using one way ANOVA followed by Dunnett's test p < 0.05, p < 0.0001 was considered as statistically significant.

## Funding source

None.
