# Supplementary material for: Data of in vivo screening of antiulcer activity for methanolic extract of Vernonia elaeagnifolia DC
Source: Data Brief. 2019 Mar 7;23:103753. doi: 10.1016/j.dib.2019.103753 (PMC6681831; doi:10.1016/j.dib.2019.103753)
Supplement: Supplementary file 1 — Multimedia component 1 [file mmc1.docx]

Conflict of Interest and Authorship Conformation Form

Please check the following as appropriate:

- All authors have participated in (a) conception and design, or analysis and interpretation of the data; (b) drafting the article or revising it critically for important intellectual content; and (c) approval of the final version.
- This manuscript has not been submitted to, nor is under review at, another journal or other publishing venue.
- The authors have no affiliation with any organization with a direct or indirect financial interest in the subject matter discussed in the manuscript
- The following authors have affiliations with organizations with direct or indirect financial interest in the subject matter discussed in the manuscript:

Author’s name Affiliation

Dr. Sneha Ramesh Nawale Associate Prof., Department of Pharmacognosy,

Gokaraju Rangaraju College of Pharmacy,

Bachupally, Hyderabad-500090

N. Priyanka Student (M.Pharmacy) , Gokaraju Rangaraju College of Pharmacy, Bachupally, Hyderabad-500090.

Sujit Das Student (M.Pharmacy) , Gokaraju Rangaraju College of Pharmacy, Bachupally, Hyderabad-500090.

Dr. M. Gangaraju Professor and HOD, Department of Pharmacology,

Gokaraju Rangaraju College of Pharmacy, Bachupally, Hyderabad-500090.
